# The Promise of Pharmacogenomics in Reducing Toxicity During Acute Lymphoblastic Leukemia Maintenance Treatment

**DOI:** 10.1016/j.gpb.2016.11.003

**Published:** 2017-04-06

**Authors:** Shoshana Rudin, Marcus Marable, R. Stephanie Huang

**Affiliations:** 1 Biological Sciences Division, University of Chicago, Chicago, IL 60637, USA; 2 Department of Medicine, University of Chicago, Chicago, IL 60637, USA

**Keywords:** Acute lymphoblastic leukemia, 6-Mercaptopurine, Methotrexate, Pharmacogenomics, Maintenance therapy

## Abstract

Pediatric **acute lymphoblastic leukemia** (ALL) affects a substantial number of children every year and requires a long and rigorous course of chemotherapy treatments in three stages, with the longest phase, the maintenance phase, lasting 2–3 years. While the primary drugs used in the maintenance phase, **6-mercaptopurine** (6-MP) and **methotrexate** (MTX), are necessary for decreasing risk of relapse, they also have potentially serious toxicities, including myelosuppression, which may be life-threatening, and gastrointestinal toxicity. For both drugs, pharmacogenomic factors have been identified that could explain a large amount of the variance in toxicity between patients, and may serve as effective predictors of toxicity during the maintenance phase of ALL treatment. 6-MP toxicity is associated with polymorphisms in the genes encoding thiopurine methyltransferase (*TPMT*), nudix hydrolase 15 (*NUDT15*), and potentially inosine triphosphatase (*ITPA*), which vary between ethnic groups. Moreover, MTX toxicity is associated with polymorphisms in genes encoding solute carrier organic anion transporter family member 1B1 (*SLCO1B1*) and dihydrofolate reductase (*DHFR*). Additional polymorphisms potentially associated with toxicities for MTX have also been identified, including those in the genes encoding solute carrier family 19 member 1 (*SLC19A1*) and thymidylate synthetase (*TYMS*), but their contributions have not yet been well quantified. It is clear that **pharmacogenomics** should be incorporated as a dosage-calibrating tool in pediatric ALL treatment in order to predict and minimize the occurrence of serious toxicities for these patients.

## Introduction

Acute lymphoblastic leukemia (ALL) is the most common cancer among children [1], which specifically involves the precursors of B and T cells. Specific sets of somatic genetic alterations are conserved between all types of ALL, among both B and T cell precursors [2]. Within the United States alone, there are approximately 2900 ALL cases diagnosed annually in children and adolescents under the age of 20 [3]. ALL is more commonly diagnosed in males, and differences among ethnic groups have been observed, with Hispanic children affected more frequently than other ethnic groups [4]. While these dispositions are conserved with respect to adult ALL, adult and pediatric ALL outcomes differ, as pediatric patients have benefited more from new treatments [4]. Historically, these prognoses were rather poor with a high risk of remission, but due to significant developments in the field, overall long-term survival rates are up to 90% as reported in 2015 [5].

Complete pediatric ALL treatment is divided into three distinct periods, each with different treatment goals. Treatment begins after diagnosis with the induction phase, where physicians attempt to induce remission immediately, followed by the consolidation phase, where physicians attempt to remove any residual disease remaining after the induction phase, and finally the maintenance phase, where physicians attempt to reduce the possibility of relapse [6]. In order to fulfill the specific goal for each treatment phase, the drugs chosen and the amounts given during each period differ greatly between phases.

In the induction phase, chemotherapeutic agents including vincristine, anthracyclines, corticosteroids, and asparaginase are used to immediately kill off leukemic cells [6]. These drugs are known for high toxicity and substantial side effects. In the consolidation phase, these drugs are used in various combinations designed to maximize synergy [6]. Although the drugs used in these first two stages are relatively toxic, these phases of the treatment usually tend to last for a period of several weeks to several months only. In contrast, the maintenance phase is far longer, lasting approximately 2–3 years in pediatric patients [6]. 6-mercaptopurine (6-MP) and methotrexate (MTX) are the drugs predominantly used during maintenance therapy, which have been used consistently and investigated extensively as well. Ideally, chemotherapeutic agents used for so long a period of time should have minimal side effects, or only result in side effects of a low severity. Nonetheless, this is not the case for 6-MP and MTX, since both of them have potentially serious and life-threatening toxicities that can occur in a subset of patients [7].

Considering the pharmacogenomic effects of treating patients with drugs that have potentially varied toxicity is essential, as it may guide physicians toward identifying and minimizing the risk of life-threatening toxicity in pediatric ALL patients. Given the narrow therapeutic window of chemotherapeutic drugs, the high toxicity of the drugs must be balanced with their actual effectiveness in treating the cancer when physicians determine which drugs to prescribe and how high a dose to use [7]. This narrow therapeutic window must be factored in when considering all cancer treatments. This is particularly true when it comes to ALL treatment, since patients will be exposed to these drugs for 2–3 years during the maintenance phase. Any way to gauge for which patients toxicity may be a greater concern could help plan dosages appropriately in order to minimize those toxic effects.

The pharmacogenomics of cancer treatments usually concern itself with two separate genomes: the genome of the cancer cells and the genome of the patient’s normal tissues. The efficacy of chemotherapeutic agents at removing cancer cells can depend substantially on the cancer genome. For this reason, applying pharmacogenomic techniques to the cancer cells themselves may help determine which treatments will have an effect in curing the cancer, and which treatments might have little to no efficacy. However, efficacy of the drug can also depend on the patient’s ability to absorb and metabolize the drug, which can lead to the treatment being ineffective at actually curing the cancer, a great concern in 6-MP therapy [8]. In addition, the toxicity of chemotherapeutic agents may vary in response to alterations in the patient’s genome. 6-MP and MTX have been well-studied, and variations in their toxicities have been associated with a number of genomic variations, the details of which will be explained in this review.

## ALL treatment protocols

Survival rates for pediatric ALL have improved substantially in recent years; however, the ultimate goal in ALL treatment is to have the best survival outcomes with the least toxicity. As such, treatment regimens are typically designed aiming to minimize toxicity for the patient while maximizing survival.

**Figure 1:**
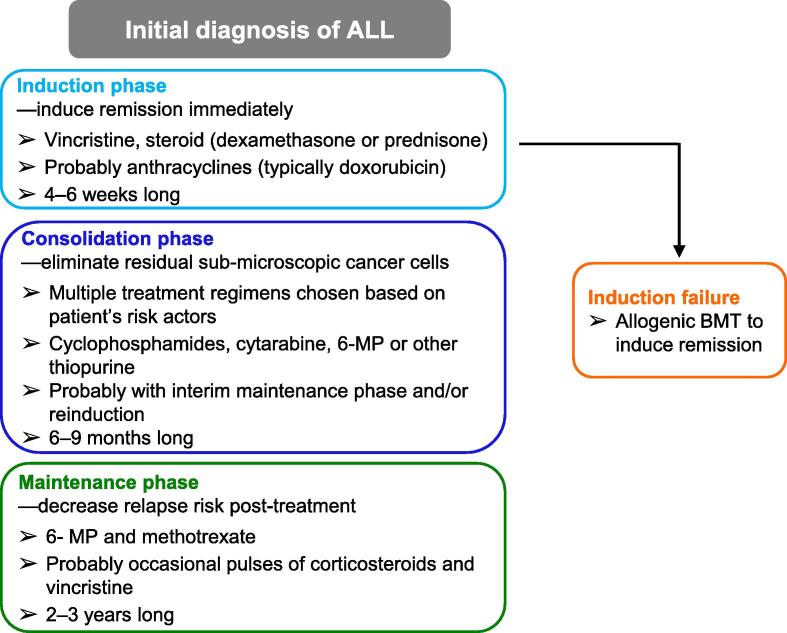
**Diagram of the treatment phases of pediatric acute lymphocytic leukemia**

Immediately post-diagnosis, the induction phase of ALL treatment begins with a multi-drug regimen, typically 3 or 4 drugs (Figure 1). All induction protocols include vincristine, either dexamethasone or prednisone, and L-asparaginase, whereas many protocols also include an anthracycline, typically doxorubicin [9]. The Children’s Oncology Group (COG) typically reserves the use of the 4-drug regimen including anthracyclines for T-cell and high-risk B-cell-precursor ALL, and the use of the 3-drug regimen without anthracyclines for normal-risk B-cell-precursor ALL [9]. These drugs are given with the intent of aggressively eliminating leukemic cells in order to induce complete remission. In order to achieve remission in patients with more difficult-to-treat cancers or in patients who have relapsed, allogeneic bone marrow transplants can be performed in the event of induction failure [5].

Once complete remission has been achieved, the therapies used are modified slightly to prevent relapse and eliminate any submicroscopic cancer that may be left (Figure 1). Various risk factors for a given patient are taken into account, resulting in variations in the dosage and drug regimen for the consolidation phase of treatment [9]. Higher risk patients receive a higher intensity level of consolidation treatment than patients who are at lower risk, which improves their outcomes substantially [10]. The most commonly-used Berlin–Frankfurt–Münster (BFM) consolidation regimen consists of four phases: an initial consolidation phase including cyclophosphamides, cytarabine, and 6-MP (or another thiopurine); an interim maintenance phase comprising primarily MTX; a reinduction phase typically with many drugs used the same as those during induction and the initial consolidation phase; and finally, entrance into the maintenance phase of treatment [9]. More aggressive intensification during the maintenance phase of treatment is associated with much higher toxicity [10]. However, the intensification of treatment is still essential in order to prevent relapse.

The maintenance phase of treatment is the final and longest stage of treatment (Figure 1), mainly focused on anti-metabolite therapy [5]. Typically, 6-MP is given orally on a daily basis, with MTX given orally or parenterally on a weekly basis [9]. The maintenance regimen may also include occasional pulses of corticosteroids and vincristine [5]. Outcomes decrease substantially when the maintenance phase is shortened even by six months, with a reported increase in relapses under those conditions [11]. Substantial critical pharmacogenomic concerns have been raised concerning the efficacy and the toxicity of 6-MP and MTX. Given that patients are treated with these drugs for 2–3 years, it is essential to investigate pharmacogenomics of the drugs used in this phase in order to avoid substantial toxicity and poor outcomes.

## Pharmacogenetic considerations for 6-MP

**Figure 2:**
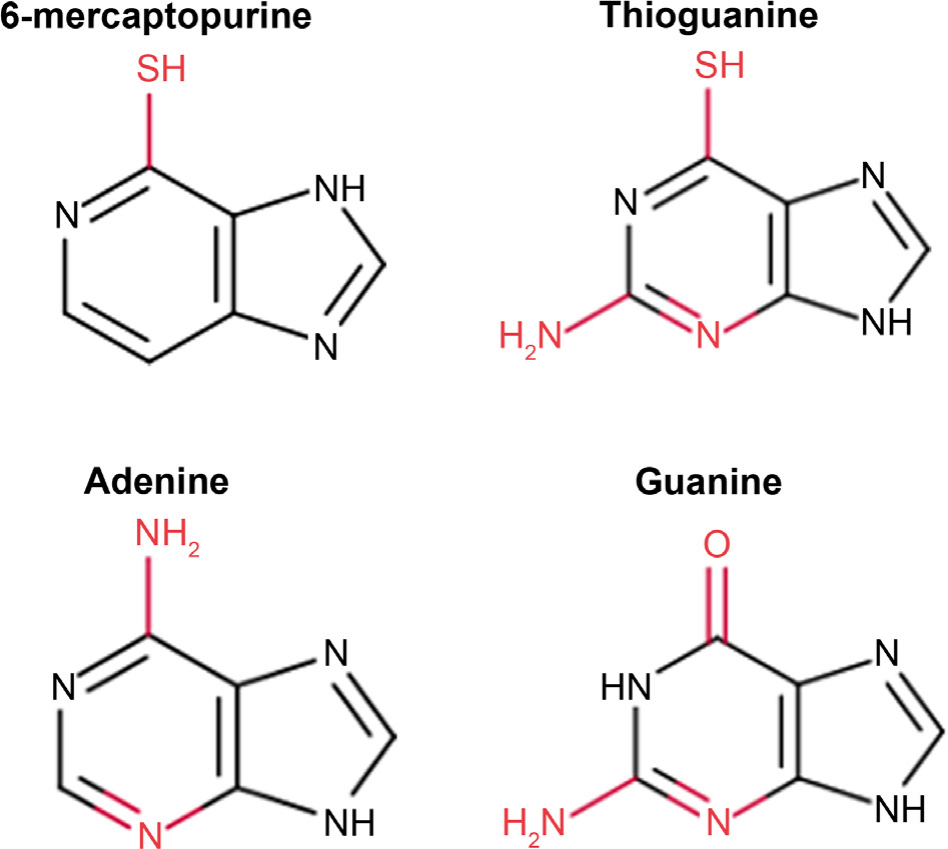
**Structures of 6-MP and thioguanine in comparison to adenine and guanine** Thiopurines are metabolized into thiopurine nucleotides that substitute regular adenine and guanine nucleotides, leading to cytotoxicity in cells treated with those drugs. The key differences between each molecule are shown in red. 6-MP, 6-mercaptopurine.

**Figure 3:**
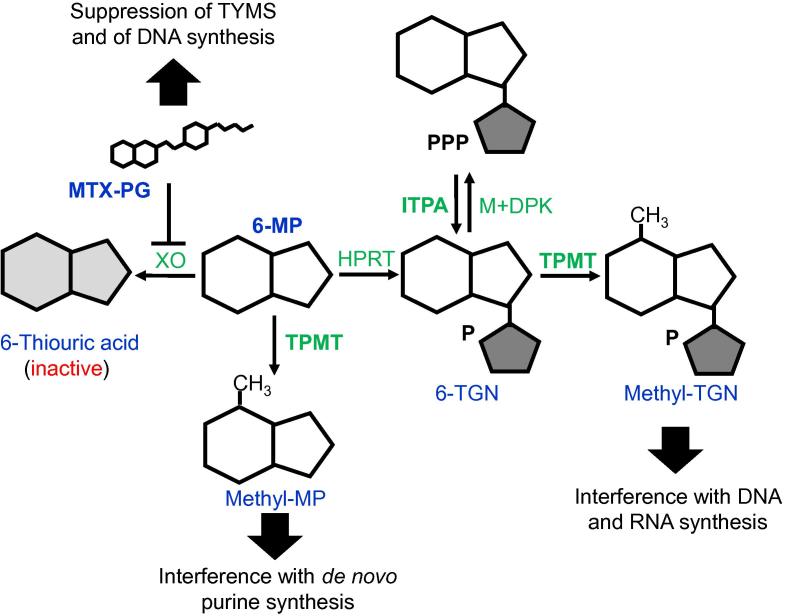
**6-MP metabolism** 6-MP is converted by HPRT to 6-TGNs, which are methylated by TPMT. Methyl-TGNs block DNA and RNA synthesis. 6-MP is also methylated directly by TPMT, producing methylmercaptopurine (methyl-MP). MTX-PG blocks the conversion of 6-MP to its inactive metabolite thiouric acid. MTX-PG, methotrexate polyglutamate; 6-MP, 6-mercaptopurine; TGN, thioguanine nucleotide; TPMT, thiopurine methyltransferase; ITPA, inosine triphosphatase; TYMS, thymidylate synthetase; HPRT, hypoxanthine phosphoribosyl transferase; DPK, diphosphate kinase; XO, xanthine oxidase.

6-MP is a thiopurine drug mimicking the chemical structure of physiologically-occurring purines (Figure 2). Like thioguanine (also shown in Figure 2), 6-MP is metabolized into thiopurine nucleotides [12], which are incorporated into DNA during cell replication and ultimately stop replication and lead to cytotoxicity. 6-MP is used as one of the two primary drugs during the maintenance phase of ALL and has been a component of ALL therapy at this stage for over 40 years [13]. The metabolism of 6-MP into thiopurine nucleotides, such as methyl-MP and methyl-thioguanine nucleotide (methyl-TGN), involves several enzymes (Figure 3). 6-MP is converted by hypoxanthine phosphoribosyl transferase (HPRT) into its active form, MP nucleotides, which are collectively referred to as 6-TGNs [7]. 6-TGNs are then methylated by thiopurine methyltransferase (TPMT), producing methyl-TGNs that interfere with DNA and RNA synthesis, leading to cytotoxicity [7]. Additionally, TPMT methylates 6-MP directly, converting it to methylmercaptopurine, which interferes with *de novo* purine synthesis [7], [12].

There are known genetic variants of the *TPMT* gene, which can result in an increased risk of myelosuppression, a particularly serious toxicity associated with 6-MP therapy [13]. Untreated myelosuppression can lead to bone marrow failure; as such, it is important to determine what genetic variants increase the risk of toxicity during 6-MP therapy [13], [14]. Genetic polymorphisms leading to reduced TPMT enzyme activity are associated with a substantially higher risk of myelosuppression [15]. The vast majority of TPMT deficiency can be attributed to four variant alleles: TPMT*2 (238G>C), *3A (460G>A, 719A>G), *3B (460G>A), and *3C (719A>G) (Table 1), although copy number variations and deletions of exons also account for the variability in TPMT efficacy to some extent [15]. These variant alleles lead to decreased activity of *TPMT*, and result in a higher risk of toxicity. *TPMT* expression may also be influenced by environmental factors, as evidenced by the increased expression during thiopurine treatment and decreased expression with age and renal malfunction [16]. In addition, variants in linkage disequilibrium with the most common *TPMT* single nucleotide polymorphism (SNP), 719A>G (rs1142345), have also been connected to altered *TPMT* expression [16]. Genome-wide association study (GWAS) analysis demonstrates that *TPMT* polymorphisms *3A and *3C have the strongest associations with toxicity. For example, SNP 719A>G results in an amino acid change from tyrosine (Y) to cysteine (C) with a slight dose-dependent effect, whereas patients with homozygous GG genotype at this site displayed the greatest decrease in 6-MP dose tolerance [17]. All major *TPMT* polymorphisms (*2, *3A, *3B, and *3C) are of greatest prevalence in patients of European and African descent, but are incredibly rare in patients of East Asian descent [17]. Despite the risk of myelosuppression, shortening the course of 6-MP results in a far higher risk of relapse [18]. Therefore, clinical efforts to avoid myelosuppression must also balance the necessary dosage and length of 6-MP course needed to achieve desirable therapeutic effects. *TPMT* genotyping has already been performed in patients in various institutions, both preemptively and in the presence of signs of myelosuppression [13], [15]. Nonetheless, *TPMT* is not the only gene that may affect 6-MP toxicity. Other genes, in particular, *PACSIN2*, *MRP4*, *ITPA* (shown in Figure 3), and *NUDT15* have also been investigated.

*PACSIN2* encodes the protein kinase c and casein kinase substrate in neurons 2, which plays a role in intracellular vesicle-mediated transportation and caveolae formation. PACSIN2 is thought to be associated with TPMT activity and its effects on 6-MP [19], [20], [21]. GWAS analysis initially identified *PACSIN2* polymorphisms, especially rs2413739 (NC_000022.10:g.43397036C>T), as a significant determinant of TPMT activity, which was later supported by clinical trials [20], [22]. Even after controlling for TPMT activity, this *PACSIN2* SNP was still significantly associated with gastrointestinal toxicity in ALL patients treated with 6-MP [20]. However, such association was not replicated in patients with inflammatory bowel syndrome after treatment with thiopurines [23].

*ABCC4* encodes multiple drug resistance protein 4 (MRP4), which belongs to the ATP-binding cassette (ABC) transporter family and assists in the efflux of a large variety of therapeutic agents, including 6-MP [24]. Of the 11 missense variants that have been investigated, compared to the wild-type MRP4, the Y556C variant has been associated with significantly higher transport of 6-MP, whereas the V776I, and the rs9516519 T>G polymorphisms have been associated with significantly lower transport 6-MP [25]. As MRP4 protects against 6-MP toxicity by transporting its metabolites out of the cells, the identification of these variants in patients could yield useful information when recommending a treatment regimen, if coupled with other pharmacogenomic relationships that exacerbate 6-MP toxicity [26].

*ITPA* and *NUDT15* have been proposed as additional candidate genes with potential pharmacogenetic effects during 6-MP treatment. *ITPA* codes for inosine triphosphatase that catalyzes the hydrolysis of inosine triphosphate to inosine monophosphate, thus preventing the buildup of harmful nucleotides in cells [19]. Genetic polymorphisms in *ITPA*, specifically 198C>A, cause low ITPA activity and may also be associated with a higher risk of myelosuppression [19]. *NUDT15* is yet another possible candidate affecting 6-MP toxicity risks. *NUDT15* encodes nudix hydrolase 15, which also helps in the removal of damaged nucleotides from the cell. The *NUDT15* SNP 415C>T (rs116855232) is associated strongly with thiopurine-induced leukopenia [27], [28]. Further studies have also reported the association of *NUDT15* 415C>T with general toxicity and 6-MP induced myelosuppression [28], [29], [30], [31].

Variant alleles of *ITPA* and *NUDT15* are more common, while *TPMT* variants are too rare to explain many cases of 6-MP toxicity in East Asian, native American, and Hispanic populations. Therefore, *ITPA* and *NUDT15* may be better candidates for 6-MP pharmacogenomics in these populations [19], [27]. For instance, GWAS analysis reveals significant associations between *NUDT15* 415C>T and myelosuppression, especially in the aforementioned populations. Among Taiwanese children with ALL, 11.6% harbored the *NUDT15* risk allele, while only 1.6% carried a risk allele for *TPMT* [17]. The frequency of the *NUDT15* risk allele was 9.8% in East Asians, 3.9% in Hispanics, and 0.2% in Europeans, whereas only the wild type allele was found in Africans [17]. The same study also demonstrated significance of *TPMT* and *NUDT15* but not *ITPA* risk alleles with decreased dose tolerance for 6-MP [17]. Taken together, these studies suggest that *NUDT15* may be a better candidate for future investigations when trying to search for other variants of clinical significance [17].

## Pharmacogenetic considerations for MTX

**Figure 4:**
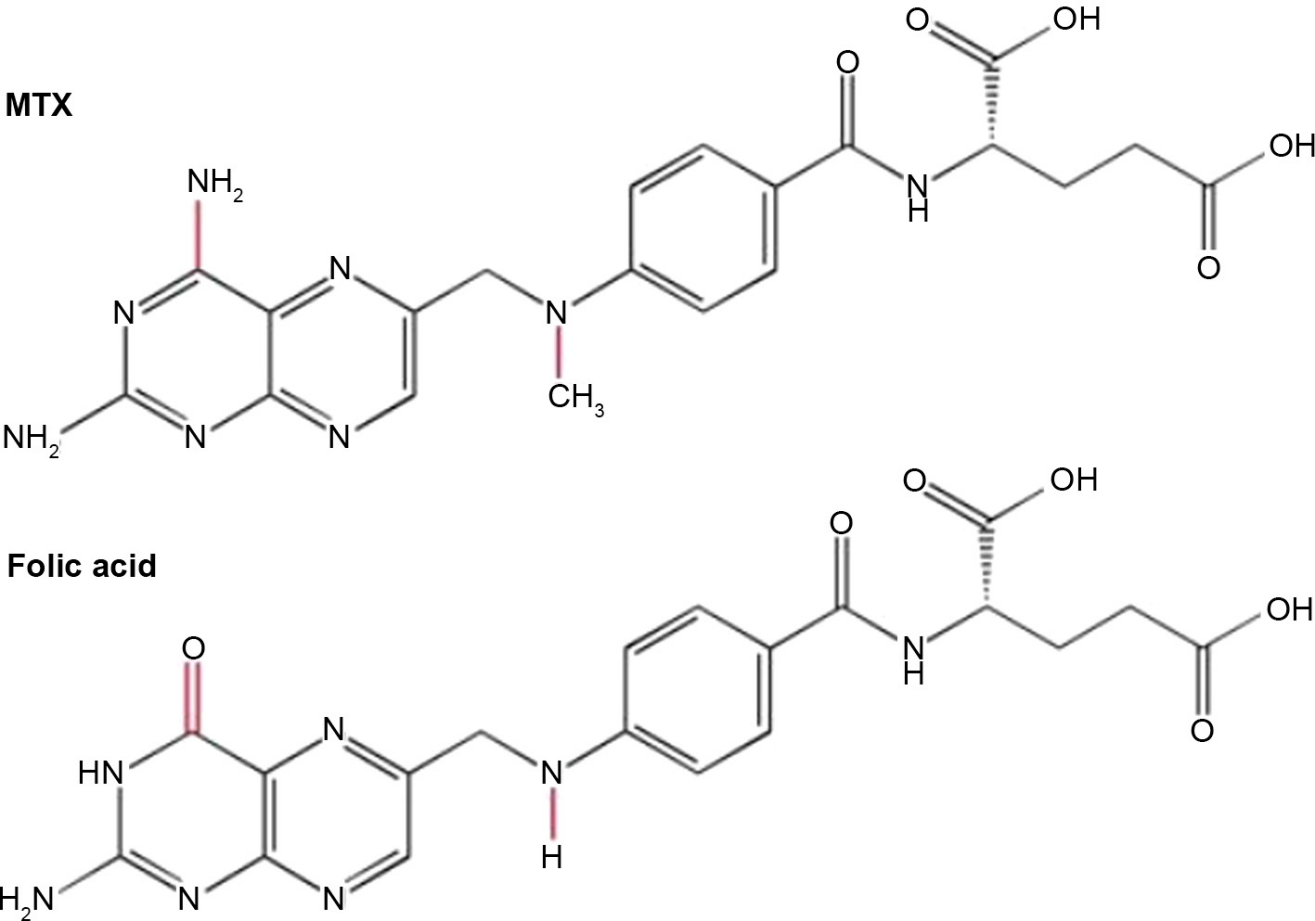
**Structures of MTX and folic acid** The molecular structures of MTX and folic acid. The key differences between MTX and folic acid are shown in red. MTX, methotrexate.

MTX was introduced into clinical practice for the first time in the 1950 s and has become one of the drugs commonly used during maintenance therapy for ALL [32]. In order to understand the specific ways through which MTX function can be altered by genomic variants, it is necessary to understand mechanism of action of MTX. MTX is extremely similar to folic acid or folate in terms of structure (Figure 4). Thereby, MTX acts as a competitive inhibitor of enzymes that utilize folate and possess a 1000-fold increased affinity for these enzymes [32].

**Figure 5:**
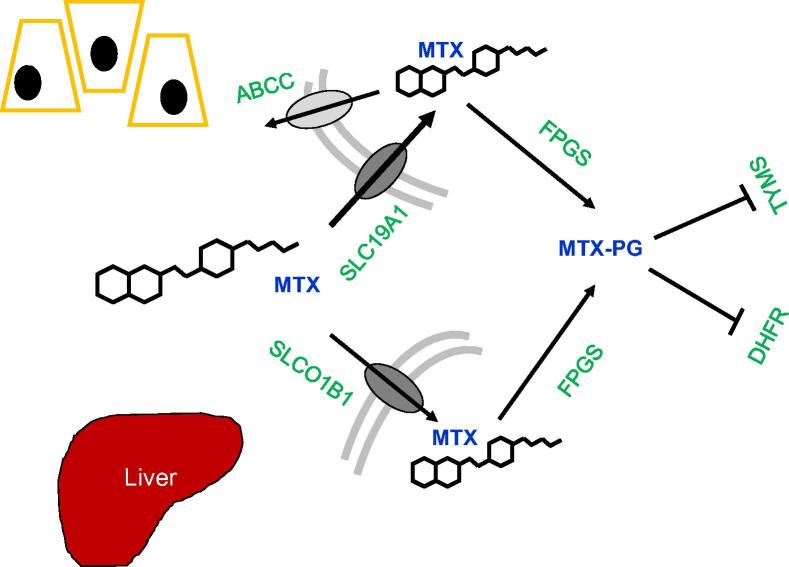
**Cellular pathway and action of MTX** MTX enters cells through SLC19A1 and SLCO1B1 transporters, and leaves cells through the ABCC protein. Inside cells, MTX is converted by FPGS into its active form, MTX-PG, which then inhibits TYMS and DHFR. SLCO1B1 is almost exclusively found on hepatocytes in the liver. MTX, methotrexate; MTX-PG, methotrexate polyglutamate; SLC19A1, solute carrier family 19 member 1; SLCO1B1, solute carrier organic anion transporter family member 1B1; ABCC, ATP-binding cassette transporter subfamily C; FPGS, folylpolyglutamate synthase; TYMS, thymidylate synthetase; MTHFR, methylenetetrahydrofolate reductase; DHFR, dihydrofolate reductase.

MTX enters cells mainly through the reduced folate carrier 1 (RFC-1), also known as the solute carrier family 19 member 1 (SLC19A1) transport protein. There are also other significantly less utilized transport proteins, such as the solute carrier organic anion transporter 1B1 (SLCO1B1) that is largely located on human hepatocytes (Figure 5) [33]. Once inside the cells, MTX is converted into its active form of MTX polyglutamate (MTX-PG) under the catalyzation by folylpolyglutamate synthetase (FPGS). MTX-PG competitively inhibits the dihydrofolate reductase (DHFR), which would otherwise catalyze an essential reaction for DNA precursor synthesis, to suppress DNA and RNA synthesis [33]. It also inhibits the thymidylate synthase (TYMS) that converts dUMP to dTMP, providing an additional pathway to suppress DNA and RNA synthesis [13]. In order for MTX to be metabolized, the ABC sub-family C (ABCC), an efflux MTX transporter, is needed for MTX removal from cells [33]. As would be expected for any drug acting on such an integral cellular process, many enzymes are involved in this pathway that can potentially affect drug efficacy and thus warrant further study.

Studies that have sought to uncover the underlying pharmacogenomic relationships of MTX had reported varied findings. Several mutations in SLC19A1 have been found to confer a general resistance to MTX. Mutations located within the transmembrane domain of RFC-1, including R27H, G44E, E45 K, S46 N, I48F, and W105G, diminish the ability of RFC-1 to situate itself within the cell membrane, which have been correlated with the diminished capacity of RFC-1 to transport MTX [34], [35], [36], [37], [38], [39], [40]. The strongest evidence is available for the R27H mutation. Several recent studies have associated the R27H mutation with increased MTX plasma concentration and toxicity, although no *RFC-1* polymorphism has been found to significantly affect MTX response in GWAS analyses [41], [42], [43]. It is known that MTX responses differ between ethnic groups, especially between Asian and Caucasian populations [44]. Given the lack of significance discovered for *RFC-1* polymorphisms, significant attention has only been paid to R27H or 80G>A [44]. Other than *RFC-1*, the rs11045879 and rs4149081 polymorphisms of *SLCO1B1*, which are localized to hepatocyte membranes, have been correlated with gastrointestinal toxicity and quicker drug clearance. These two hyperactive SLCO1B1 variants result in almost exclusive transport of MTX to the liver, where it then passes into the gastrointestinal tract, rendering it severely underactive and leading to MTX clearance [45]. The hyperactive variants are believed to be fairly rare across all ethnicities, but there are observed differences in the allele frequency for the underactive variant 521T>C [46]. It is of note that these alleles are believed to have little to no presence in the African and Oceanic populations, whereas the allele frequencies are about 15% and 24% in the Middle Eastern and Caucasians, as well as in the South and Central American populations, respectively [46].

Methylenetetrahydrofolate reductase (MTHFR), an enzyme that acts to catalyze reactions within the methyl cycle and *de novo* thymidine synthesis, has been researched due to the ability of MTX-PG to interfere with MTHFR expression. The MTHFR variants, while being among the most studied of MTX related groups, have been found to have a diverse range of effects. Some studies on pediatric patients reported that the 677C>T variant of *MTHFR* was associated with relapse, whereas no association was revealed between the 1298A>C variant and relapse, toxicity, or infection [47]. Additional studies reported that the 677C>T variant has been associated with neurotoxicity and liver toxicity [48]. Therefore, 677C>T and 1298A>C variants have been argued to be useful in monitoring the neurotoxicity and liver toxicity of patients undergoing ALL maintenance therapy [49]. However, other studies reported that these variants have no significant effect on protein function of MTHFR [50]. It should be pointed out that the clinical trials which failed to establish any association between these variants and a given effect were performed using only 53 patients [50].

The real effect of *TYMS* gene polymorphisms is similarly questionable. Repeats of the *TYMS* gene enhance both the expression and activity of the TYMS protein. However, whether these repeats result in MTX or MTX-PG resistance is still up for discussion, as separate studies have shown that triple tandem repeats of the *TYMS* gene have ambiguous relationships with ALL relapse [51], [52].

The SNP 829C>T in *DHFR*, which occurs near the miR-24 binding site, causes a general elevation of *DHFR* expression. Unlike *MTHFR* and *TYMS*, the elevated expression of *DHFR* is well known to be correlated with a resistance to MTX and MTX-PG [13]. Finally, the rs3740065 A>G polymorphism in *ABCC2* has been found to cause significantly-reduced ABCC activity [53]. As expected, this variant leads to a significantly increased level of MTX and MTX-PG present within the cell, subsequently causing toxicity and resistance [53]. The effect of *ABCC* gene polymorphisms can be recapitulated by the up-regulation of miR-453, which decreases the activity of several ABCC family proteins [13].

Given that several of these genomic variants are associated with severe toxicity, that other variants are associated with robust resistance to MTX, and that MTX treatment is designed to last for years, a patient’s polymorphisms must be taken into account when prescribing MTX treatment.

## Other pharmacogenomic issues in pediatric ALL treatment

Ongoing research efforts on the pharmacogenomics of pediatric ALL during the maintenance phase are not limited to the aforementioned drugs. For instance, allopurinol in conjunction with MP treatment has been proposed. Allopurinol directs the metabolism of 6-MP toward its functional form of 6-TGN, thus diverting MP away from its toxic derivatives [54]. However, there are pharmacogenomic considerations for allopurinol, since the incidence of severe cutaneous adverse reactions induced by allopurinol is significantly increased in the presence of HLA-B*58:01, encoding the human leukocyte antigen (HLA) B58 that plays a critical role in the immune system [55]. Therefore, when considering this combinatorial treatment, it is important to acknowledge that while the introduction of allopurinol does force physicians to consider the presence of certain gene variants, there is a tradeoff for the consideration of 6-MP. With the addition of allopurinol, less 6-MP would be required for ALL treatment. Several studies have indicated fewer instances of pancreatitis in patients undergoing 6-MP treatment when treated with allopurinol [56]. This tradeoff could be significantly advantageous for certain individuals, depending on their genotype.

Other drugs that have already been used regularly during the treatment of pediatric ALL include L-asparaginase, glucocorticoids, and vincristine, all of which have potential pharmacogenomic concerns as well. L-asparaginase is commonly used during the induction phase of ALL treatment. Its use is associated with serious adverse effects that span across multiple organ systems, such as allergic reactions, pancreatitis, and cerebrovascular accidents. These serious adverse effects occur in up to 45% of patients with life-threatening anaphylaxis occurring in 10% of patients experiencing adverse effects [13]. SNPs in the gene encoding asparaginase synthetase (ASNS), which catalyzes the transfer of an amino group onto aspartic acid, can both positively and negatively affect the gene expression of *ASNS* [13]. However, whether higher *ASNS* expression necessarily correlates to a superior therapeutic efficacy of L-asparaginase is still a contested topic. Other genes, such as *AMPA1* that encodes the ionotropic glutamate receptor and *HLA-DRB* that encodes a portion of the HLA complex antigen-binding dimer, have been studied more thoroughly and have more established relationships with asparaginase. Several SNPs in *AMPA1* and HLA-DRB*0701 have been correlated with a hypersensitivity to asparaginase treatment and a subsequent incidence of allergy following L-asparaginase treatment [57], [58].

Glucocorticoids are an essential component to ALL therapy. Given their pivotal role in the treatment of pediatric ALL, potential pharmacogenomic relationships have been aggressively pursued. There is correlation between glucocorticoid resistance and the upregulation of the *ABCB1* gene, specifically the 3435C>T, 2677G>T/A, and 129T>C *ABCB1* variants, whereas *IL-10* upregulation increases sensitivity to glucocorticoids [59], [60], [61], [62]. In addition, *GST* deletion has been associated with increased initial response to glucocorticoids and severity of infectious complications by decreasing glucocorticoid metabolism [63].

Vincristine can cause mitotic arrest and leukemic cell death by binding to tubulin dimers and interfering with spindle fiber formation. It is capable of inducing neurotoxicity, which can manifest in a variety of ways, such as constipation and motor sensory dysfunction [13]. The neurotoxicity is one of the most serious and unpredictable problems with pediatric ALL patients. CYP3A5 is known to metabolize approximately 60% of vincristine present in the body [13]. However, studies have failed to confirm that SNPs in *CYP3A5* are responsible or even correlated with the variable degrees of neurotoxicity, leaving room for a significant amount of further investigations [64].

All of the examples above demonstrate the relevance of pharmacogenomic testing in determining dosage in pediatric ALL therapy. In each of these cases, efficacy or toxicity may be affected by various pharmacogenomic components. Ideally, risk alleles related to all drugs in the planned course of chemotherapy should be assessed prior to beginning treatment for these patients, which would be far more efficient than testing for one risk allele at a time, or at the beginning of each phase of treatment. As vincristine and glucocorticoids may be incorporated into the maintenance phase of treatment and allopurinol could be used to optimize 6-MP treatment, a comprehensive pharmacogenomic panel for maintenance therapy, at minimum, should include alleles relevant to these drugs, as well as 6-MP and MTX.

The influences of the drugs above are not limited to their individual drug–gene interactions, but also involve the relationships between these drugs. These drug–drug interactions can lead to the subsequent exacerbation of the effects that specific genetic variants have on a particular patient. The most relevant instance of drug–drug interactions combined with pharmacogenomic interactions occurs during the maintenance phase between 6-MP and MTX (Figure 3) [65]. MTX inhibits xanthine oxidase (XO) [66], which catalyzes the transformation of 6-MP into its inactive form, 6-thiouric acid. By inactivating XO, MTX increases the throughput of 6-MP into its active form, 6-TGN, thus augmenting its effect. Under such situation, mutations in *TPMT* or *HPRT* would be exacerbating, as they are the only remaining functional enzymes involved in catalyzing 6-MP.

## Clinical applications

The clinical applications of pharmacogenomics in the maintenance phase of pediatric ALL treatment are substantial. For 6-MP therapy, recommendations for pharmacogenomic testing have already been implemented. Recommendations regarding *TPMT* testing are already in place, with hospitals such as St. Jude Children's Research Hospital testing all ALL patients for *TPMT* variants prior to starting 6-MP therapy in order to better titrate dosage [13]. The US Food and Drug Administration (FDA) has also issued a recommendation that patients undergo genetic testing for *TPMT* variation prior to beginning therapy with 6-MP [55]. Clinical recommendations have been put forward suggesting that, whether based on phenotype of TPMT activity and signs of myelosuppression or preemptive *TPMT* genotyping, 6-MP dosage should be adjusted depending on a patient’s TPMT activity in order to reduce the risk of myelosuppression (Table 2).

While the FDA has not yet released any statements regarding testing for *ITPA* or *NUDT15* variants prior to 6-MP therapy, such testing may be beneficial. Testing for *NUDT15* polymorphisms may be particularly advantageous in Taiwanese and other East Asian patients, given the low prevalence of toxicity-associated *TPMT* alleles in this population and the much higher frequency of the *NUDT15* risk allele [27]. East Asian patients actually have a higher risk of 6-MP-related toxicities, making it compelling for further *NUDT15* testing in these patients [27]. On the other hand, *ITPA*-related risks of myelosuppression have been less convincingly demonstrated, therefore testing for those variants may not yet be clinically warranted [17].

The gene–drug interactions of MTX have not yet been well characterized as for 6-MP. Consequently, the FDA has not yet made any recommendations with regard to pharmacogenetic testing prior to MTX therapy. However, some testing may well be warranted. *SLCO1B1* variants have already been well studied to have an effect on MTX toxicity [45]. Testing for those *SLCO1B1* variants associated with increased risk of MTX toxicity may become a part of clinical pharmacogenomics in pediatric ALL treatment in the near future. The effects of *DHFR* polymorphisms are not well quantified yet, so genotyping *DHFR* in patients is not yet clinically relevant. Nonetheless, as more information is obtained, this may also become important in medical practice. Similarly, there is not a substantial body of evidence to point conclusively toward the clinical importance of genotyping for risk alleles in *MTHFR*, *TYMS*, *ABCC2*, and *ABCC4*. As more studies look into these genes, their clinical relevance may suggest the need for clinical genotyping as well.

## Conclusion

While there are significant gaps in knowledge with respect to the various pharmacogenomic relationships in pediatric ALL, in recent years, larger and more comprehensive studies have elucidated much concerning the drugs used during the maintenance period of pediatric ALL treatment. 6-MP is by far the most explored element of the maintenance period. Sufficient evidence has been found demonstrating the diminishing effect that TPMT*2, *3A, *3B, and *3C allele variants have on TPMT protein levels, and subsequent increased risk of myelosuppression. Consequently, in 2015 the FDA officially issued a recommendation for genetic testing for these alleles prior to therapy. While other genes such as *ITPA* and *NUDT15* have been similarly revealed to affect the risk of myelosuppression, none of them have been investigated as thoroughly as *TPMT*. MTX has no FDA-approved genomic testing prior to use, as the enzymes involved in its metabolism and translocation have been studied less vigorously and found to affect far fewer individuals. However, certain genomic variants found in genes such as *RFC-1*, *SLCO1B1*, *DHFR*, and *ABCC* can be said clearly to have a detrimental effect on MTX function, resulting in toxicity or lack of efficacy. Other drugs involved in pediatric ALL treatment have been similarly explored, with the most attention being directed toward glucocorticoids due to their necessity for ALL therapy and significant potential disadvantages. While no FDA approval has been issued as of yet, many genes have been discovered to affect glucocorticoid response.

For both 6-MP and MTX, the evidence that we have outlined in this article suggests that pharmacogenomic testing should become a part of dosage criteria when treating patients with these drugs. Body surface area and other traditional measures for drug dosage should be accompanied by genotyping for the specific genes that we have outlined here, due to these many genetic differences affecting metabolism of these drugs. The therapeutic necessity of using these drugs is clear. Despite the risks of toxicity with certain genotypes, both of these drugs are necessary to decrease risk of relapse in pediatric ALL treatment. This indicates that the predominant clinical relevance of such information should be in dosage. While a certain length of the treatment course appears to be necessary, the dosage of 6-MP and MTX should be carefully calibrated using all information available including pharmacogenomic data, in order to maximize therapeutic effect while minimizing the risks of potentially dangerous toxicity. As a result of trustworthy sources becoming more numerous, clinical actions are being shaped. Hospitals, such as St. Jude Children's Research Hospital, have already begun addressing the problem of prescribing the necessary dosage, while simultaneously avoiding unneeded toxicities by genotyping patients that are prescribed 6-MP, specifically looking for *TPMT* variants [16]. However, other hospitals primarily genotype for *TPMT* following the onset of symptoms of myelosuppression and clinical practices surrounding treatment with MTX that incorporate pharmacogenomics have not yet been developed.

Going forward, we propose several recommendations for the incorporation of the pharmacogenomics of MTX and 6-MP into pediatric ALL treatment. Preemptive genotyping for *TPMT* and *NUDT15* variants should be performed prior to 6-MP therapy, with consideration given to the patient’s ethnicity when deciding which of the two variants takes priority. Due to the potentially life-threatening toxicity of 6-MP, patients homozygous for the risk allele must be identified prior to the start of 6-MP therapy, so that dosage could be adjusted to prevent severe myelosuppression. Given the lesser degree of evidence regarding MTX pharmacogenomics, patients experiencing gastrointestinal toxicity and reduced MTX efficiency should be genotyped for *SLCO1B1* variants, while those experiencing other toxicities may warrant genotyping of other genes discussed in this review. On a per-patient basis, dosage should be adjusted as needed to reflect a patient’s putative risk alleles.

Additional studies of MTX pharmacogenomics are also needed. Ideally, given further research and more definitive evidence of which alleles are of greatest concern in MTX treatment, a pharmacogenomic panel of alleles relevant to all of the drugs used in maintenance therapy, potentially including optional drugs such as vincristine and glucocorticoids, should be made readily available in hospitals in order to optimize the availability and efficiency of pharmacogenomic testing prior to pediatric ALL maintenance therapy. The patient’s genotype should be considered in conjunction with other clinically-relevant information in order to select a dosage of these drugs that will be maximally effective while minimizing the risk of toxicity over the 2–3 years of maintenance therapy. These recommendations, if put into practice consistently, should help to reduce the toxicity risks associated with the long course of this phase of ALL treatment.

## Competing interests

The authors have declared no competing interests.

## Figures and Tables

**Table 1:** **Summary of genetic polymorphisms involved in 6-MP and MTX metabolism**

**Gene**	**Polymorphisms**	**dbSNP ID**	**Variation type**	**Possible consequences**	**Refs.**
*DHFR*	829C>T	rs5030762	UTR	MTX resistance	[13]

*TPMT*	*2: 238G>C	rs1800462	A80P	Myelosuppression when treated with 6-MP	[13], [15]
	*3A: 460G>A, 719A>G		A154T, Y240C		
	*3B: 460G>A	rs1800460	A154T		
	*3C: 719A>G	rs1142345	Y240C		

*ASNS*	≥2 repeats; 181C>T			Increased L-asparaginase therapeutic efficacy	[13]

*ITPA*	198C>A	rs1127354	P32T	Higher risk of myelosuppression	[19]

*PACSIN2*	C>T	rs2413739	Intronic	Gastrointestinal toxicity when treated with 6-MP	[19], [20], [21]

*ABCC4*	A>G		Y556C	Increased 6-MP clearance	[24], [25], [26]
	G>A		V776I	Decreased 6-MP clearance	
	T>G	rs9516519	UTR	MTX toxicity and resistance	[53]

*NUDT15*	415C>T	rs116855232	R139C	Thiopurine-induced leukopenia	[27], [28]

*RFC-1*	80G>A	rs1051266	R27H	Diminished MTX transported into the cell	[34], [35], [36], [37], [38], [39], [40]
			G44E, E45K, S46N, I48F, and W105G		

*SLCO1B1*	T>C	rs11045879	Intronic	Gastrointestinal toxicity	[45], [46]
	T>C	rs4149081	Intronic	Quick drug clearance	
	521T>C	rs4149056	V174A		

*MTHFR*	677C>T	rs1801133	A222V	Relapse, neurotoxicity, liver toxicity	[47], [48], [49], [50]
	1298A>C	rs1801131	E429A		

*TYMS*	2 repeats; 3 repeats			MTX resistance; ALL relapse	[51], [52]

*ABCC2*	A>G	rs3740065	Intronic	Decreased MTX flux; MTX toxicity and resistance	[53]

*AMPA1*	G>A	rs4958351	Intronic	L-asparaginase hypersensitivity and allergy	[57], [58]
	C>T	rs10070447	Intronic		

*HLA-DRB*	*0701			L-asparaginase hypersensitivity and allergy	

*ABCB1*	3435C>T	rs1045642	Synonymous	Glucocorticoid resistance	[59], [60], [61], [62]
	2677G>T/A	rs2032582	A893S/T		
	129T>C	rs3213619	UTR		

*CYP3A5*	6986A>G	rs776746	Intronic	Neurotoxicity as a response to vincristine treatment	[13], [64]
	14690G>A	rs10264272	Synonymous		

*Note*: DHFR, dihydrofolate reductase; TPMT, thiopurine methyltransferase; ASNS, asparaginase synthetase; ITPA, inosine triphosphatase; PACSIN2, protein kinase C and casein kinase substrate in neurons 2; NUDT15, nudix hydrolase 15; RFC-1, reduced folate carrier 1; SLCO1B1, solute carrier organic anion transporter family member 1B1; MTHFR, methylenetetrahydrofolate reductase; TYMS, thymidylate synthetase; ABC, ATP-binding cassette; AMPA1, ionotropic glutamate receptor; HLA-B, major histocompatibility complex, class I, B; CYP, cytochrome P450; 6-MP, 6-mercaptopurine; MTX, methotrexate; ALL, acute lymphoblastic leukemia.

**Table 2:** **Clinical recommendations for 6-MP dosage based on *TPMT* genotype and phenotype of ALL patients**

***TPMT* genotype**	**TPMT phenotype**	**Risks of 6-MP therapy**	**Dosage recommendations**
Homozygous wild-type	Normal to high enzyme activity	Minimal risk of myelosuppression	Follow standard dosing procedures
Heterozygous	Intermediate enzyme activity	Increased risk of myelosuppression	Begin with starting dose 30%–70% of normal, adjust as needed

Homozygous for TPMT*2, *3A, *3B, and *3C	Low or no enzyme activity	High risk of potentially life-threatening myelosuppression	Begin with starting dose reduced tenfold and reduce frequency of doses, adjust as needed

*Note*: Data were summarized based on evidence and recommendations listed in Relling et al. [66].
